# Incidence, survival comparison, and novel prognostic evaluation approaches for stage iii-iv pulmonary large cell neuroendocrine carcinoma and small cell lung cancer

**DOI:** 10.1186/s12885-023-10797-3

**Published:** 2023-04-05

**Authors:** Liling Huang, Yu Feng, Tongji Xie, Haohua Zhu, Le Tang, Yuankai Shi

**Affiliations:** Department of Medical Oncology, National Cancer Center/National Clinical Research Center for Cancer/Cancer Hospital, Chinese Academy of Medical Sciences & Peking Union Medical College, Beijing Key Laboratory of Clinical Study On Anticancer Molecular Targeted Drugs, No. 17 Panjiayuan Nanli, Chaoyang District, Beijing, 100021 China

**Keywords:** Pulmonary large cell neuroendocrine carcinoma, Small cell lung cancer, SEER database, High-grade neuroendocrine carcinomas, Lung cancer

## Abstract

**Background:**

Pulmonary large cell neuroendocrine carcinoma (LCNEC) and small cell lung cancer (SCLC) are two types of high-grade neuroendocrine carcinomas of the lung with poor prognosis. LCNEC has not been thoroughly studied due to its rarity, data are also lacking regarding the survival comparison and prognosis analysis of patients with locally advanced or metastatic LCNEC and SCLC.

**Methods:**

Data of patients with LCNEC, SCLC, and other NSCLC who were diagnosed from 1975 to 2019 were extracted from the Surveillance, Epidemiology and End Results (SEER) database to estimate incidence. Those in stage III-IV and being diagnosed from 2010 to 2015 were utilized further to investigate their clinical characteristics and prognosis. Propensity score matching (PSM) analyses at a ratio of 1:2 was used to compare their survival outcomes. Nomograms of LCNEC and SCLC were established with internal validation, and the nomogram of SCLC was externally validated by 349 patients diagnosed in Cancer hospital, Chinese Academy of Medical Sciences & Peking Union Medical College from January 1, 2012 to December 31, 2018.

**Results:**

The incidence of LCNEC has been increasing in recent decades, meanwhile that of SCLC and other types of NSCLC were decreasing. A total of 91,635 lung cancer patients, including 785 with LCNEC, 15,776 with SCLC, and 75,074 with other NSCLC were enrolled for further analysis. The survival of stage III-IV LCNEC resembles that of SCLC, and significantly worse than other types of NSCLC before and after PSM analysis. In pretreatment prognostic analysis, age, T stage, N stage, M stage, bone metastasis, liver metastasis, and brain metastasis were found to be associated with the survival of both LCNEC and SCLC, besides sex, bilaterality, and lung metastasis were additional prognostic factors for SCLC. Two nomograms and convenient online tools respectively for LCNEC and SCLC were established accordingly with favorable predicting accuracy of < 1-year, < 2-year, < 3-year survival probabilities. In external validation of the SCLC nomogram with a Chinese cohort, the AUCs of 1-year, 2-year and 3-year ROC were 0.652, 0.669, and 0.750, respectively. All the results of 1-, 2-, 3- year variable-dependent ROC curves verified the superior prognostic value of our nomograms for LCNEC and SCLC over the traditional T/N/M staging system.

**Conclusions:**

Based on large sample-based cohort, we compared the epidemiological trends and survival outcomes between locally advanced or metastatic LCNEC, SCLC, and other NSCLC. Furthermore, two prognostic evaluation approaches respectively for LCNEC and SCLC might present as practical tools for clinicians to predict the survival outcome of these patients and facilitate risk stratification.

**Supplementary Information:**

The online version contains supplementary material available at 10.1186/s12885-023-10797-3.

## Introduction

According to the 2015 World Health Organization (WHO) classification of lung tumors, pulmonary large cell neuroendocrine carcinoma (LCNEC) and small cell lung cancer (SCLC) are two types of high-grade neuroendocrine carcinomas of the lung with poor prognosis, which composed of 2–3% and 15% of lung cancer [1–5]. Regarding LCNEC is a rare kind of disease, it is not thoroughly studied and well characterized as other NSCLC and SCLC, most of the studies of LCNEC are of retrospective nature and in small sizes. Though LCNEC occupies a small proportion of lung cancer, it has received more attention in recent years due to better understanding of this distinct identity and its unfavorable prognosis. Both LCNEC and SCLC are more susceptible to elderly male heavy smokers with 5-year overall survival (OS) rates of 15–25% and < 15%, respectively [3]. Approximately two thirds of patients with SCLC were initially diagnosed in extensive stage, 40–70% of LCNEC were initially diagnosed in stage III-IV [3, 6, 7]. LCNEC has a high incidence of recurrence after surgery, even in stage I disease [3], while for those in stage III-IV, the 5-year OS rates were nearly zero [3, 8].

LCNEC could be located in peripheral or midzone, while most of SCLC are in central position. One of the main histology differentiation diagnoses of LCNEC and SCLC is based on cell size and nuclear features, while sometimes it might be confusing for pathologists [2]. In terms of molecular pattern, LCNEC presents as a distinct subgroup of lung cancer, which could be categorized into “type I LCNEC” and “type II LCNEC”: type I LCNEC shares similar genomic alterations (STK11/KEAP1 alterations) with other types of NSCLC, while the transcriptional characteristics and neuroendocrine profile are shown more resemblance to SCLC. Type II LCNEC is more similar to SCLC in genetics (with RB1 alterations), but with lower levels of neuroendocrine markers [9]. Varlotto et al. proposed that the clinical and biological features of LCNEC are more similar to other large cell carcinomas than to SCLC [10], while some studies found that the clinical features of early-stage LCNEC are more like other types of NSCLC, while advanced LCNEC resembles SCLC in survival outcomes and metastatic pattern [7, 11].

In this study, we focused on patients with locally advanced and metastatic (stage III-IV) LCNEC and SCLC, tried to investigate the clinical characteristics, survival, and prognosis of this population based on the latest data of the Surveillance Epidemiology and End Results (SEER) database, aiming to have a deeper understanding of high-grade neuroendocrine lung carcinomas, and to develop respective prognostic evaluation approaches to facilitate risk stratification of LCNEC and SCLC.

## Methods

### Database and participants

A first cohort of lung cancer patients diagnosed between January 1, 1975 and December 31, 2019 which were extracted from 8 registries of the SEER database was used to estimate the epidemiological trend of long-term age-adjusted incidence of lung cancer including two specific histology types (i.e. LCNEC and SCLC).

A second cohort of patients with stage III-IV LCNEC, SCLC, and other NSCLC who were diagnosed between January 1, 2010 and December 31, 2015 were extracted from 17 registries of the SEER database for analyses of clinical characteristics, survival, and prognosis. The histology types of lung cancer were identified according to the International Classification of Diseases for Oncology, 3rd Edition (ICD-O-3): i) LCNEC (ICD-O-3 8013), ii) SCLC (ICD-O-3 8041–8045), and iii) other NSCLC (ICD-O-3 8010, 8012, 8020, 8021, 8046, 8050, 8051, 8052, 8070–8076, 8140, 8141, 8250–8255, 8260, 8310, 8430, 8480, 8481, 8490, 8560, 8570, 8571, 8574, 8575). The primary cancer sites were restricted on lung and bronchus (site code: C34.0-C34.3 and C34.8-C34.9). Exclusion criteria included: without confirmed histology; with unknown information regarding clinical variables that included in this study; with unknown survival status or survival time = 0; have more than one primary tumor. The detailed process of SEER data acquisition and filtration were summarized in Additional file 1. The whole SEER data we used in this study can be accessed in Additional file 2.

Additionally, a third cohort of 349 patients with stage III-IV SCLC who were diagnosed from January 1, 2012 to December 31, 2018 were collected from Cancer hospital, Chinese Academy of Medical Sciences & Peking Union Medical College, a top-class hospital in China, for validation. This cohort has been approved by the ethics committee/institutional review board at Cancer hospital, Chinese Academy of Medical Science & Peking Union Medical College.

### Study variables

Clinical variables including incidence, age, sex, race, primary site, bilaterality, T/N/M stage, organ metastases (bone/brain/liver/lung metastases), treatment strategies (surgery, radiation, and chemotherapy), survival time, and survival status were extracted. For continuous variable “age”, its optimal cutoff value (70 years old) was calculated using the “surv_cutpoint” function of “survminer” R package. “Bilaterality” refers to the situation that patients has tumors on both side of the lung. The TNM stage was according to the 7th edition of American Joint Committee on Cancer (AJCC) TNM staging system. OS was defined as the time from randomization to the date of death of any cause or the last follow-up.

### Incidence trend analysis

The incidence of lung cancer (per 100 000 patients per year) and annual percentage changes (APCs) were calculated using SEER*Stat software (version 8.4.0) and under age-adjustment to the 2000 U.S. standard population. The survival curves were generated using Kaplan–Meier curves by log-rank test.

### PSM analysis to compare survival of LCNEC, SCLC, and other NSCLC

Nearest-neighbor propensity score matching (PSM) analyses between LCNEC and SCLC, and between LCNEC and other NSCLC respectively both at a ratio of 1:2 was applied to balance potential baseline covariates including age, sex, race, bilaterality, TNM stage, bone/brain/liver/lung metastases status and treatment strategies, and to compare the survival outcomes of three subgroup histology types.

### Nomogram development and validation

To investigate the valid pretreatment prognostic factors of stage III-IV LCNEC and SCLC, all enrolled LCNEC and SCLC patients from the SEER database were randomly divided into the training and validation cohorts at a ratio of 7:3, respectively. In the training cohort, prognostic analysis was conducted by univariate and multivariable Cox regression analysis, the variables with *P* < 0.05 in univariable analyses were selected into a step-wise multivariable analysis to identify independent risk factors. Then the data was used for the establishment of nomogram prognostic models for LCNEC and SCLC, respective. Two separate nomograms for LCNEC and SCLC predicting < 1-year, < 2-, and < 3-year OS rates were constructed based on the data of respective training cohorts. Validation cohort and external validation cohort (if have) were used for internal and external verification of the established nomograms. Calibration curves were constructed to verify the extent of consistence between the predicted and actual survival. 1-, 2-, and 3-year receiver operating characteristic (ROC) curves and variable-dependent ROC curves was generated to assess the accuracy of prognostic significance of the nomograms. Decision curve analysis (DCA) was conducted to evaluate the net clinical benefits of the nomograms. “surv_cutpoint” function of “survminer” R package was utilized again to divide patients into relatively low-risk and high-risk according to the individual score calculated by nomograms.

### Establishment of online tools

Two online nomogram tools of LCNEC and SCLC were established for automatically realizing patient nomogram score calculation, risk group determination, and survival probability prediction via R and the Shiny website (https://www.shinyapps.io/).

### Statistical analysis

All analyses were performed via R software (version 4.1.0) using survival, survminer, rms, regplot, MatchIt, DynNom, Shiny, and ggplot2 R-packages. A two-side *P*-value < 0.05 was considered statistically significant.

## Results

### Annual incidence

**Fig. 1:**
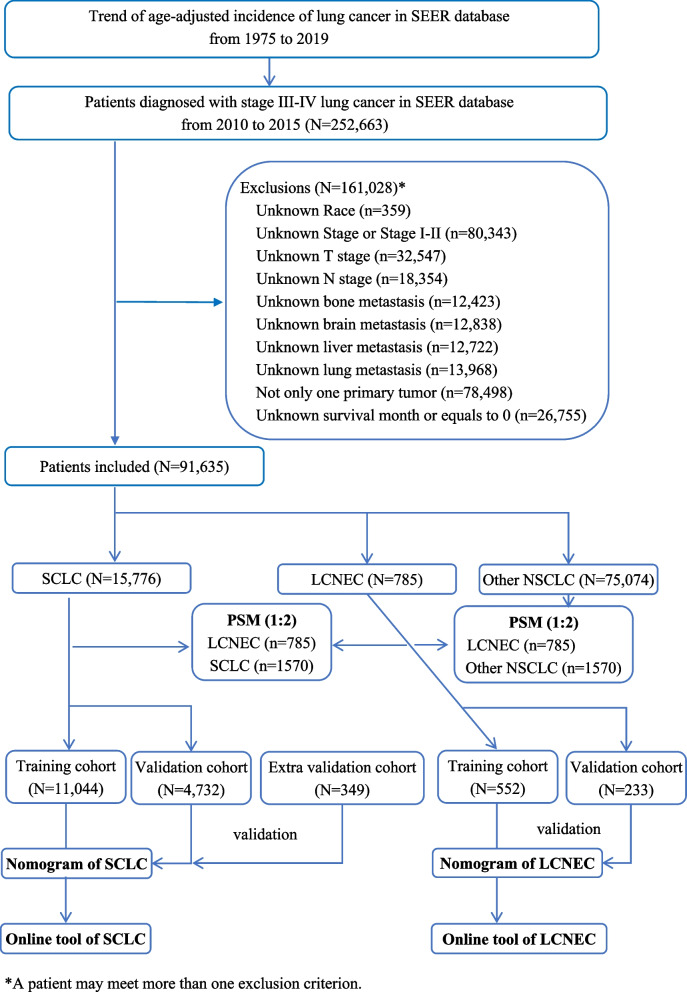
Study flow diagram. Abbreviation: LCNEC, large cell neuroendocrine carcinoma; SCLC, small cell lung cancer; NSCLC, non-small cell lung cancer; OS, overall survival; SEER, the Surveillance Epidemiology and End Results; PSM, propensity score matching

**Fig. 2:**
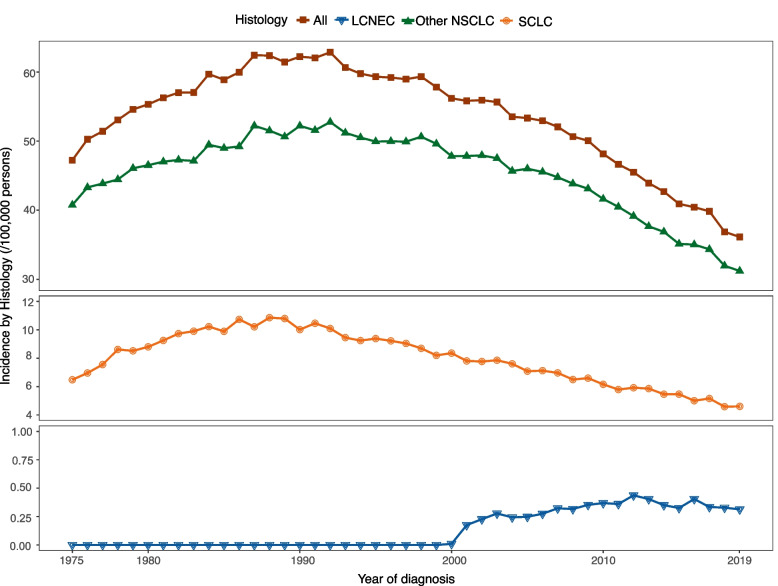
Annual age-adjusted incidence of lung cancer, LCNEC, SCLC, and other types of NSCLC. Abbreviation: LCNEC, large cell neuroendocrine carcinoma; SCLC, small cell lung cancer; NSCLC, non-small cell lung cancer; OS, overall survival

The flow diagram of this study is presented in Fig. 1. The data of population in SEER 8 registries from 1975 to 2019 were utilized for analyzing the incidence of whole lung cancer, two specific histology types (i.e. LCNEC and SCLC), and others types of NSCLC. The annual age-adjusted incidence of lung cancer patients increased from 47.24 per 100 000 persons in 1975 to 62.85 per 100 000 persons in 1992, while has decreased since then to 36.13 per 100 000 persons in 2019. The APC for age-adjusted incidence from 1975 to 2019 was -0.87 per 100 000 persons (95%CI: -1.12 ~ -0.61, *P* < 0.05). For patients who were diagnosed with LCNEC, the annual age-adjusted incidence was 0 per 100 000 persons in 1975–1999 and increased to 0.31 per 100 000 persons in 2019. For patients with SCLC, the annual age-adjusted incidence was 6.48 per 100 000 persons in 1975, which increased to 10.86 per 100 000 persons in 1988 and then decreased annually to 4.61 per 100 000 persons in 2019 with an APC of -1.57 per 100 000 persons (95%CI -1.94 ~ -1.21, *P* < 0.05). The trend of annual age-adjusted incidences of all lung cancer, LCNEC, SCLC, and other types of NSCLC from 1975 to 2019 were presented in Fig. 2.

### Patients characteristics

**Fig. 3:**
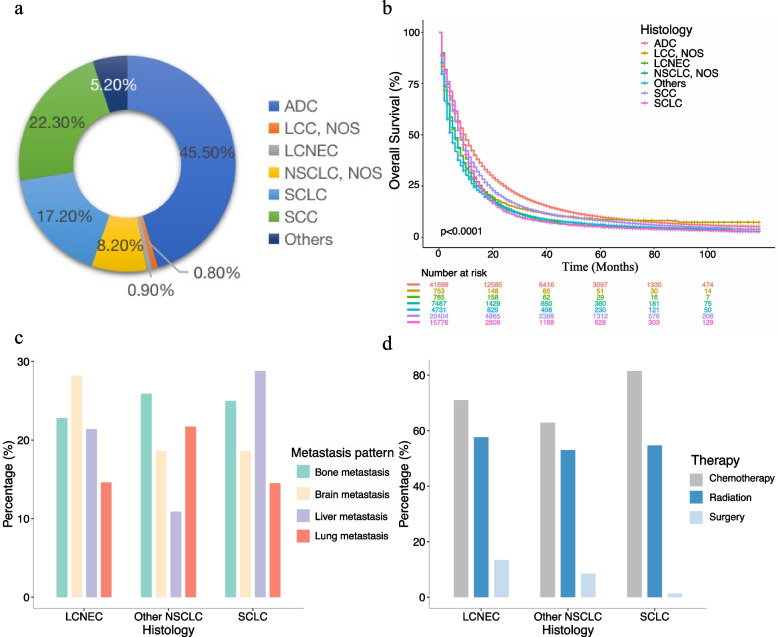
The comparison of clinical characteristics of stage III-IV LCNEC, SCLC, and other types of NSCLC. **a** the proportion of different subtypes of lung cancer. **b** K-M curves of different histologies of lung cancer. **c** metastatic pattern. **d** treatment strategies. Abbreviation: LCNEC, large cell neuroendocrine carcinoma; ADC, adenocarcinoma; SCC, squamous cell carcinoma; LCC, NOS, large cell carcinoma, not otherwise specified; SCLC, small cell lung cancer; NSCLC: non-small cell lung cancer

A total of 91,635 patients with stage III-IV lung cancer in SEER 17 registries from 2010 to 2015 were enrolled in our analysis, which included 785 LCNEC, 15,776 SCLC, and 75,074 other types of NSCLC. The basic characteristics including age, sex, race, bilaterality, T/N/M stage, organ metastases, treatment strategies, and survival outcomes of three types of patients were summarized in Table 1. Patients who were over 70 years old accounted for 31.8%, 36.3%, and 43.1% of LCNEC, SCLC, and other NSCLC. 71.0% of LCNEC patients were first diagnosed in stage IV, followed by 69.9% of SCLC and 67.6% of other NSCLC. As shown in Fig. 3a, adenocarcinoma (45.5%) and squamous cell carcinoma (SCC) (22.3%) remained as the two predominant histology types of lung cancer, which was followed by SCLC (17.2%), while LCNEC occupied 0.9% of lung cancer. The K-M curves of detailed histologies of lung cancer were depicted in Fig. 3b. In organ metastasis (Fig. 3c), liver metastasis was most frequently observed in SCLC patients (28.8%), brain metastasis was most frequently observed in patients with LCNEC (28.2%), lung metastasis was most frequently observed in patients with other NSCLC (22.3%), while bone metastasis served as the most frequent distant metastasis site in lung cancer, and it occurred in 25.9%, 25%, and 22.8% of patients with other NSCLC, SCLC, and LCNEC, respectively. In terms of treatment (Fig. 3d), 71.0%, 57.6%, and 13.4% of patients with stage III-IV LCNEC respectively received the treatment of chemotherapy, radiation, and surgery. Meanwhile patients with SCLC received a relatively higher ratio of chemotherapy (81.5%) and a lower proportion of surgery (1.3%) than patients with LCNEC and other NSCLC, besides 54.7% of SCLC patients received radiation therapy.

**Table 1:** Patients characteristics

Variables	LCNEC	SCLC	Other NSCLC	Overall
	**(*N* = 785)**	**(*N* = 15776)**	**(*N* = 75074)**	**(*N* = 91635)**
**Age**				
< 70	535 (68.2%)	10051 (63.7%)	42718 (56.9%)	53304 (58.2%)
≥ 70	250 (31.8%)	5725 (36.3%)	32356 (43.1%)	38331 (41.8%)
**Sex**				
Female	331 (42.2%)	7859 (49.8%)	33645 (44.8%)	41835 (45.7%)
Male	454 (57.8%)	7917 (50.2%)	41429 (55.2%)	49800 (54.3%)
**Race**				
Black	102 (13.0%)	1451 (9.2%)	9651 (12.9%)	11204 (12.2%)
Others	31 (3.9%)	719 (4.6%)	6945 (9.3%)	7695 (8.4%)
White	652 (83.1%)	13606 (86.2%)	58478 (77.9%)	72736 (79.4%)
**Stage**				
III	228 (29.0%)	4744 (30.1%)	24316 (32.4%)	29288 (32.0%)
IV	557 (71.0%)	11032 (69.9%)	50758 (67.6%)	62347 (68.0%)
**T stage**				
T0	11 (1.4%)	207 (1.3%)	624 (0.8%)	842 (0.9%)
T1	113 (14.4%)	1667 (10.6%)	8685 (11.6%)	10465 (11.4%)
T2	220 (28.0%)	3895 (24.7%)	20667 (27.5%)	24782 (27.0%)
T3	185 (23.6%)	3523 (22.3%)	19624 (26.1%)	23332 (25.5%)
T4	256 (32.6%)	6484 (41.1%)	25474 (33.9%)	32214 (35.2%)
**N stage**				
N0	155 (19.7%)	1572 (10.0%)	14948 (19.9%)	16675 (18.2%)
N1	69 (8.8%)	949 (6.0%)	6363 (8.5%)	7381 (8.1%)
N2	395 (50.3%)	9431 (59.8%)	38767 (51.6%)	48593 (53.0%)
N3	166 (21.1%)	3824 (24.2%)	14996 (20.0%)	18986 (20.7%)
**M stage**				
M0	228 (29.0%)	4744 (30.1%)	24316 (32.4%)	29288 (32.0%)
M1	557 (71.0%)	11032 (69.9%)	50758 (67.6%)	62347 (68.0%)
**Bilaterality**				
No	778 (99.1%)	15654 (99.2%)	74032 (98.6%)	90464 (98.7%)
Yes	7 (0.9%)	122 (0.8%)	1042 (1.4%)	1171 (1.3%)
**Bone metastasis**				
No	606 (77.2%)	11830 (75.0%)	55663 (74.1%)	68099 (74.3%)
Yes	179 (22.8%)	3946 (25.0%)	19411 (25.9%)	23536 (25.7%)
**Liver metastasis**				
No	617 (78.6%)	11231 (71.2%)	66924 (89.1%)	78772 (86.0%)
Yes	168 (21.4%)	4545 (28.8%)	8150 (10.9%)	12863 (14.0%)
**Lung metastasis**				
No	670 (85.4%)	13482 (85.5%)	58797 (78.3%)	72949 (79.6%)
Yes	115 (14.6%)	2294 (14.5%)	16277 (21.7%)	18686 (20.4%)
**Brain metastasis**				
No	564 (71.8%)	12843 (81.4%)	61145 (81.4%)	74552 (81.4%)
Yes	221 (28.2%)	2933 (18.6%)	13929 (18.6%)	17083 (18.6%)
**Surgery**				
No/Unknown	680 (86.6%)	15575 (98.7%)	68677 (91.5%)	84932 (92.7%)
Yes	105 (13.4%)	201 (1.3%)	6397 (8.5%)	6703 (7.3%)
**Chemotherapy**				
No/Unknown	228 (29.0%)	2914 (18.5%)	27863 (37.1%)	31005 (33.8%)
Yes	557 (71.0%)	12862 (81.5%)	47211 (62.9%)	60630 (66.2%)
**Radiation**				
No/Unknown	333 (42.4%)	7143 (45.3%)	35311 (47.0%)	42787 (46.7%)
Yes	452 (57.6%)	8633 (54.7%)	39763 (53.0%)	48848 (53.3%)
**Median OS** (95% CI, months)	8.0 (7.0–9.0)	9.0 (8.0–9.0)	8.0 (8.0–8.0)	8.0 (8.0–8.0)
**6-month OS rate** (95% CI)	56.7% (53.3–60.3)	61.6% (60.8–62.3)	56.9% (56.6–57.3)	57.7% (57.4–58.1)
**1-year OS rate (95% CI)**	31.3% (28.3–34.8)	32.6% (31.8–33.3)	38.4% (38–38.7)	37.3% (37–37.6)
**2-year OS rate (95% CI)**	14.6% (12.4–17.3)	13.0% (12.5–13.5)	21.7% (21.4–22)	20.1% (19.9–20.4)
**3-year OS rate (95% CI)**	8.4% (6.7–10.6)	8.1% (7.7–8.5)	14.6% (14.4–14.9)	13.4% (13.2–13.6)

*Abbreviation*: *LCNEC* large cell neuroendocrine carcinoma, *SCLC* small cell lung cancer, *NSCLC* non-small cell lung cancer, *OS* overall survival, *CI* confidence interval

### Survival

As presented in Table 1, the median OS of patients with stage III-IV LCNEC was 8.0 months (95%CI 7.0–9.0), the 6-month, 1-, 2-, 3-year OS rates were 56.7%, 31.3%, 14.6%, and 8.4%, respectively. In patients with stage III-IV SCLC, the median OS was 9.0 months (95%CI 8.0–9.0), the 6-month, 1-, 2-, 3-year OS rates were 61.6%, 32.6%, 13.0%, and 8.1%, respectively. While other NSCLC have better survival outcome than LCNEC and SCLC with a medial OS of 8.0 months (95%CI 8.0–8.0) and 6-month, 1-, 2-, 3-year OS rates of 56.9%, 38.4%, 21.7%, and 14.6%, respectively. In prognostic analysis of the whole lung cancer population, the histology of adenocarcinoma (ADC) (HR = 0.74, *P* < 0.001) and squamous cell carcinoma (SCC) (HR = 0.91, *P* = 0.008) were protective factors compared to LCNEC in multivariate Cox regression analysis, the histology of SCLC has similar survival outcome compared to LCNEC in univariate annalysis, while SCLC tend to have better survival with an unsignificant difference (HR = 0.93, *P* = 0.057). The detailed univariate and multivariate Cox regression analysis results of the whole group with locally advanced or metastatic lung cancer were presented in Additional file 3. supplementary Table 1.

### PSM analysis of LCNEC, SCLC and other NSCLC

**Fig. 4:**
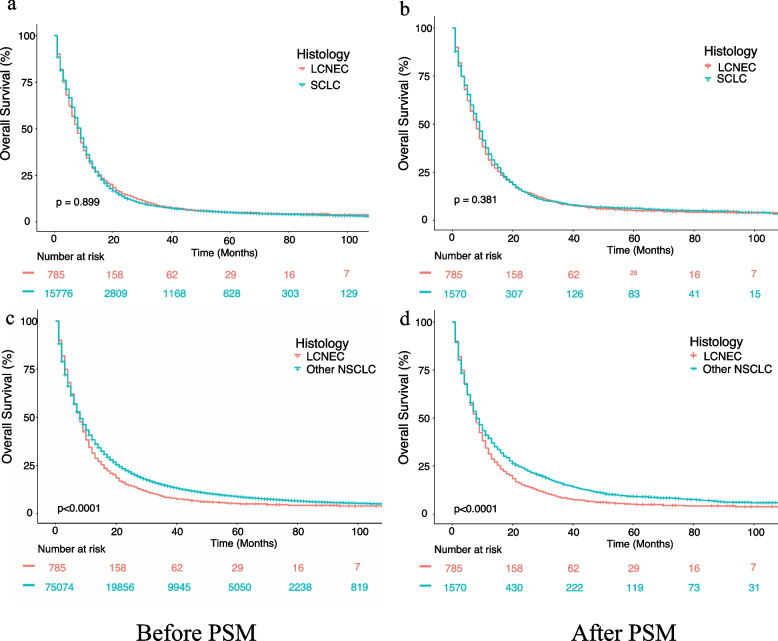
Comparison of overall survival before and after propensity score matching (PSM) analysis. **a** LCNEC and SCLC before PSM. **b** LCNEC and SCLC after PSM. **c** LCNEC and other NSCLC before PSM. **d** LCNEC and other NSCLC after PSM. Abbreviation: LCNEC, large cell neuroendocrine carcinoma; SCLC, small cell lung cancer; NSCLC, non-small cell lung cancer; OS, overall survival; PSM, propensity score matching

To further compare the survival outcome of stage III-IV LCNEC patients with that of SCLC as well as other NSCLC, we conducted a PSM analysis to match LCNEC with SCLC and other NSCLC both at a ratio of 1:2 by balancing potential covariates including age, sex, race, bilaterality, TNM stage, organ metastasis status, and treatment strategies. All covariates were well balanced after PSM (Additional file 3. supplementary Table 2–3). All patients with stage III-IV LCNEC (*n* = 785), a cohort of matched patients with stage III-IV SCLC (*n* = 1570), and a cohort of matched 1570 patients with stage III-IV other NSCLC (*n* = 1570) were included. In the comparison between LCNEC and SCLC, LCNEC and SCLC showed a similar survival outcome before PSM (Fig. 4a, log-rank *P* = 0.899) and after PSM (Fig. 4b, log-rank *P* = 0.381). In the comparison between LCNEC and other NSCLC, LCNEC was revealed to exert adverse impact on OS than other NSCLC on data before PSM and after PSM (Fig. 4c and 4d, log-rank *P* < 0.0001).

### Prognostic analysis of LCNEC, SCLC and development of nomograms

#### LCNEC

**Fig. 5:**
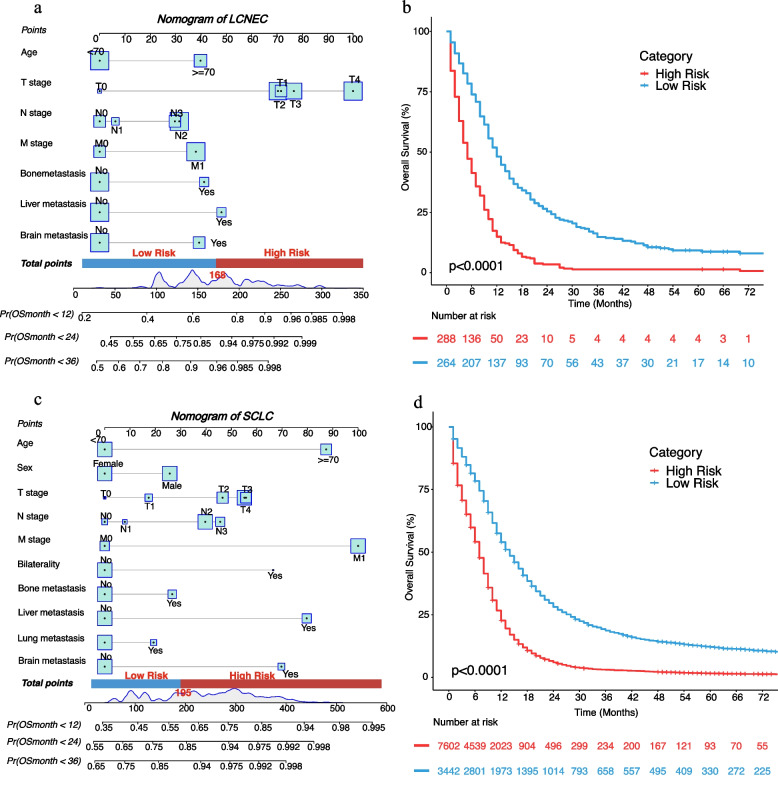
The nomograms and risk stratification of stage III-IV LCNEC and SCLC. **a** nomogram of LCNEC. **b** risk stratification of LCNEC. **c** nomogram of SCLC. **d** risk stratification of SCLC. *The cut-off point for risk stratification of LCNEC was 168 calculated by the nomogram. The cut-off point for risk stratification of SCLC was 195. Abbreviation: LCNEC, large cell neuroendocrine carcinoma; SCLC, small cell lung cancer

To explore the prognostic factors and establish a nomogram for stage III-IV LCNEC, the total 785 patients with LCNEC were randomly divided into training cohort (*n* = 552) and validation cohort (*n* = 233) in a 7:3 ratio, whose baseline characteristic were summarized in Additional file 3. supplementary Table 4. The univariate and multivariate Cox regression analyses were performed on all 552 patients with stage III-IV LCNEC in the training cohort (Table 2). The potential meaningful pretreatment variables in univariate analysis including age, T/N/M stage, bone/liver/lung/brain metastasis were selected for step-wise multivariate analysis. Finally, seven pretreatment factors were identified as independent prognostic indicators which included age, T stage, N stage, M stage, bone metastasis, liver metastasis, brain metastasis. Data of the training cohort was utilized to establish a nomogram based on the independent risk factors discovered above to predict < 1-year, < 2-year, < 3-year survival probabilities of patients with stage III-IV LCNEC. As presented in Fig. 5a, by drawing a vertical line of the value of each risk factor to the “point” horizontal line, we can obtain a score of each risk factor, then adding all scores in one patient together we can get the total nomogram score of an individual patient, with whom we can predict the survival probabilities of three time-points at the bottom of the nomogram. Of note, the risk stratification into relatively low-risk and high-risk groups was accomplished based on the score of each patient calculated by our nomogram with the cut-off point of 168 (Fig. 5b). The median OS of the high-risk and low-risk groups were 5.0 months (95%CI 4.0–6.0) and 12.0 months (95%CI 11.0–14.0), respectively. The 6-month, 1-, 2-, and 3-year OS rates of the two risk groups were also significantly different (Table 4). Notably, we developed a convenient online nomogram tool of LCNEC (https://lcnec-nomogram.shinyapps.io/dynnomapp/) to realize a multiple functions including patient nomogram score calculation, risk group determination, and survival probability prediction.

**Table 2:** Univariate and multivariate Cox analysis of patients with stage III-IV LCNEC

Variables	Univariate analysis		Multivariate analysis	
	**HR (95% CI)**	*P*	**HR (95% CI)**	*P*
**Age**				
≥ 70 vs < 70	1.32 (1.1,1.59)	0.002	1.51 (1.25,1.83)	< 0.001
**Sex**				
Male vs Female	1.12 (0.95,1.34)	0.183	-	-
**Race**				
Others vs Black	1.21 (0.72,2.04)	0.464	-	-
White vs Black	1.15 (0.89,1.48)	0.277	-	-
**T stage**				
T1 vs T0	1.67 (0.83,3.35)	0.151	2.13 (1.05,4.32)	0.036
T2 vs T0	1.93 (0.98,3.78)	0.056	2.11 (1.07,4.17)	0.032
T3 vs T0	1.95 (0.99,3.84)	0.054	2.21 (1.11,4.42)	0.024
T4 vs T0	2.22 (1.13,4.37)	0.02	2.83 (1.42,5.62)	0.003
**N stage**				
N1 vs N0	1.08 (0.76,1.53)	0.662	1.07 (0.75,1.52)	0.728
N2 vs N0	1.14 (0.91,1.42)	0.251	1.39 (1.1,1.74)	0.005
N3 vs N0	1.28 (0.98,1.68)	0.066	1.35 (1.02,1.78)	0.033
**M stage**				
M1 vs M0	1.86 (1.54,2.26)	< 0.001	1.46 (1.14,1.86)	0.002
**Stage**				
IV vs III	1.86 (1.54,2.26)	< 0.001	-	-
**Bilaterality**				
Yes vs No	0.93 (0.38,2.27)	0.87	-	-
**Bone metastasis**				
Yes vs No	2.01 (1.62,2.49)	< 0.001	1.56 (1.23,1.97)	< 0.001
**Liver metastasis**				
Yes vs No	2.11 (1.7,2.62)	< 0.001	1.64 (1.29,2.09)	< 0.001
**Lung metastasis**				
Yes vs No	1.64 (1.28,2.11)	< 0.001	1.11 (0.84,1.46)	0.459
**Brain metastasis**				
Yes vs No	1.49 (1.23,1.8)	< 0.001	1.53 (1.24,1.9)	< 0.001

*Abbreviation*: *LCNEC* large cell neuroendocrine carcinoma, *HR* hazard ratio, *CI* confidence interval

#### SCLC

Same methods were applied to patients with stage III-IV SCLC (*n* = 17,601), which was randomly divided into training cohort (*n* = 11,044) and validation cohort (*n* = 4,732) in a 7:3 ratio. The detailed univariate and multivariate results in the training cohort were demonstrated in Table 3. After multivariate Cox analysis, age, sex, T stage, N stage, M stage, bilaterality, bone metastasis, liver metastasis, lung metastasis, and brain metastasis were deemed as independent pretreatment prognostic indicators. The nomogram of stage III-IV SCLC was developed based on the training set of SCLC (Fig. 5c). The risk stratification of SCLC was achieved with cut-off points of 195 via the nomogram, and all SCLC patients were divided into low-risk and high-risk subgroups with cut-off nomogram scores as presented in Fig. 5d. A validation cohort (*n* = 4,732) and an independent validation cohort (*n* = 349) from Cancer hospital, Chinese Academy of Medical Sciences & Peking Union Medical College were used for internal and external verification. The baseline characteristics of the training, validation, and external validation cohort of SCLC were summarized in Additional file 3. Supplementary Table 5. The median OS of the high-risk and low-risk groups were 7.0 months (95%CI 7.0–7.0), 14.0 months (95%CI 13.0–15.0), respectively. The 6-month, 1-, 2-, 3-year OS rates of the two risk groups were also significantly different (Table 4). The online nomogram tool of SCLC (https://sclc-nomogram.shinyapps.io/dynnomapp/) can also realize the same convenient functions as that of LCNEC.

**Table 3:** Univariate and multivariate Cox analysis of patients with stage III-IV SCLC

Variables	Univariate analysis		Multivariate analysis	
	**HR (95% CI)**	*P*	**HR (95% CI)**	*P*
**Age**				
≥ 70 vs < 70	1.44 (1.38,1.5)	< 0.001	1.51 (1.45,1.57)	< 0.001
**Sex**				
Male vs Female	1.18 (1.13,1.22)	< 0.001	1.13 (1.09,1.17)	< 0.001
**Race**				
Others vs Black	1.06 (0.95,1.18)	0.339	0.96 (0.86,1.07)	0.483
White vs Black	1.09 (1.02,1.17)	0.008	1.03 (0.97,1.11)	0.323
**T stage**				
T1 vs T0	1.01 (0.84,1.21)	0.905	1.09 (0.9,1.3)	0.379
T2 vs T0	1.21 (1.02,1.44)	0.032	1.25 (1.04,1.49)	0.014
T3 vs T0	1.28 (1.08,1.53)	0.006	1.3 (1.09,1.55)	0.003
T4 vs T0	1.26 (1.06,1.5)	0.01	1.3 (1.09,1.55)	0.003
**N stage**				
N1 vs N0	0.99 (0.9,1.1)	0.877	1.04 (0.94,1.15)	0.457
N2 vs N0	1.04 (0.97,1.11)	0.268	1.2 (1.13,1.29)	< 0.001
N3 vs N0	1.12 (1.04,1.21)	0.002	1.24 (1.15,1.33)	< 0.001
**M stage**				
M1 vs M0	2.07 (1.98,2.16)	< 0.001	1.6 (1.51,1.68)	< 0.001
**Stage**				
IV vs III	2.07 (1.98,2.16)	< 0.001	-	-
**Bilaterality**				
Yes vs No	1.95 (1.56,2.43)	< 0.001	1.37 (1.09,1.71)	0.006
**Bone metastasis**				
Yes vs No	1.6 (1.53,1.68)	< 0.001	1.13 (1.08,1.19)	< 0.001
**Liver metastasis**				
Yes vs No	1.88 (1.8,1.96)	< 0.001	1.45 (1.38,1.52)	< 0.001
**Lung metastasis**				
Yes vs No	1.48 (1.41,1.57)	< 0.001	1.09 (1.03,1.16)	0.002
**Brain metastasis**				
Yes vs No	1.61 (1.53,1.69)	< 0.001	1.39 (1.32,1.46)	< 0.001

*Abbreviation*: *SCLC* small cell lung cancer, *HR* hazard ratio, *CI* confidence interval

**Table 4:** OS of different risk groups of stage III-IV LCNEC and SCLC

	**Risk Group**	**High Risk**	**Low Risk**
**LCNEC**	Median OS (95%CI, months)	5.0 (4.0—6.0)	12.0 (11.0—14.0)
	6-month OS rate (95%CI)	41.3% (36.0—47.4)	73.9% (68.7—79.4)
	1-year OS rate (95%CI)	14.9% (11.3—19.7)	48.1% (42.4—54.5)
	2-year OS rate (95%CI)	3.5% (1.9—6.4)	25.4% (20.6—31.2)
	3-year OS rate(95%CI)	1.4% (0.5—3.7)	14.8% (11.1—19.7)
**SCLC**	median (95%CI, months)	7.0 (7.0—7.0)	14.0 (13.0—15.0)
	6-month OS rate (95%CI)	54.1% (53.0—55.3)	78.4% (77.0—79.8)
	1-year OS rate (95%CI)	22.7% (21.8—23.7)	54.0% (52.4—55.7)
	2-year OS rate (95%CI)	6.0% (5.5—6.6)	28.1% (26.7—29.7)
	3-year OS rate (95%CI)	3.1% (2.7—3.5)	18.7% (17.4—20.1)

*Abbreviation*: *LCNEC* large cell neuroendocrine carcinoma, *SCLC* small cell lung cancer, *OS* overall survival, *HR* hazard ratio, *CI* confidence interval

#### Validation of nomograms accuracy of LCNEC and SCLC

**Fig. 6:**
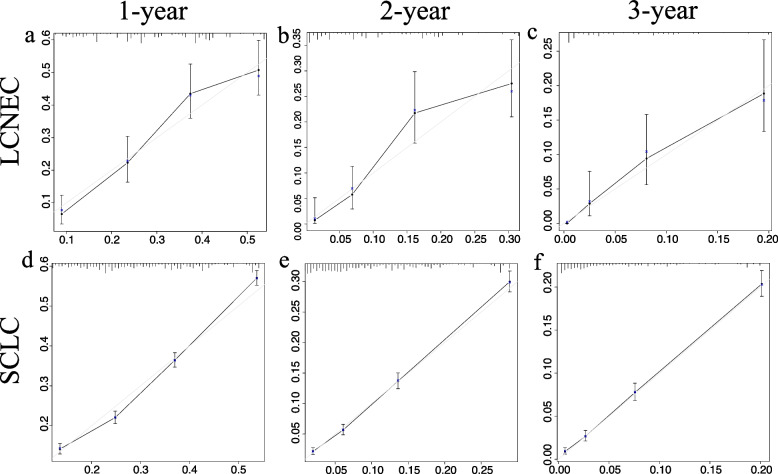
The 1-year, 2-year, and 3-year calibration plots of the training cohort and of LCNEC (**a**-**c**) and SCLC (**d-f**). Abbreviation: LCNEC, large cell neuroendocrine carcinoma; SCLC, small cell lung cancer

**Fig. 7:**
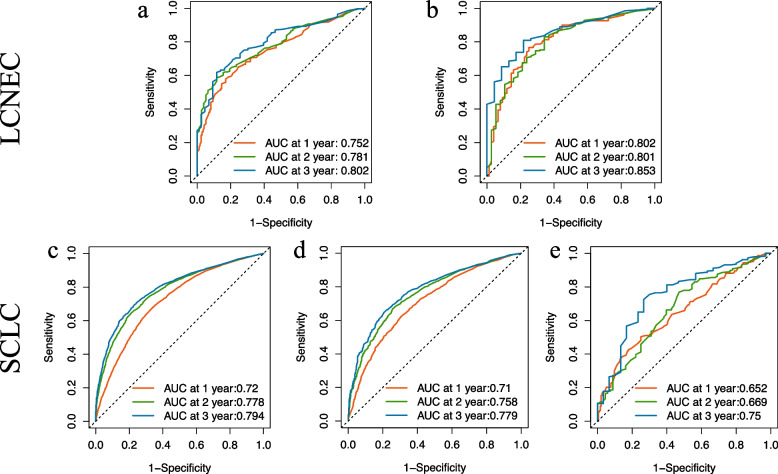
The 1-year, 2-year, and 3-year ROC curves of the training cohort and internal validation cohort of LCNEC (**a**-**b**) and SCLC (**c**-**d**), and the external validation cohort of SCLC (**e**). Abbreviation: ROC, receiver operating characteristic; AUC, area under the curve; LCNEC, large cell neuroendocrine carcinoma; SCLC, small cell lung cancer

**Fig. 8:**
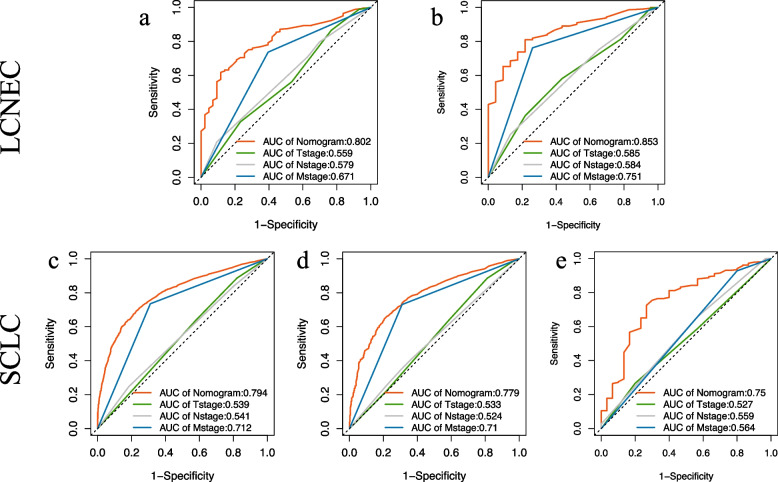
The 3-year variable-dependent ROC curves of the training cohort and internal validation cohort of LCNEC (**a**-**b**) and SCLC (**c**-**d**), and the external validation cohort of SCLC (**e**). Abbreviation: ROC, receiver operating characteristic; AUC, area under the curve; LCNEC, large cell neuroendocrine carcinoma; SCLC, small cell lung cancer

**Fig. 9:**
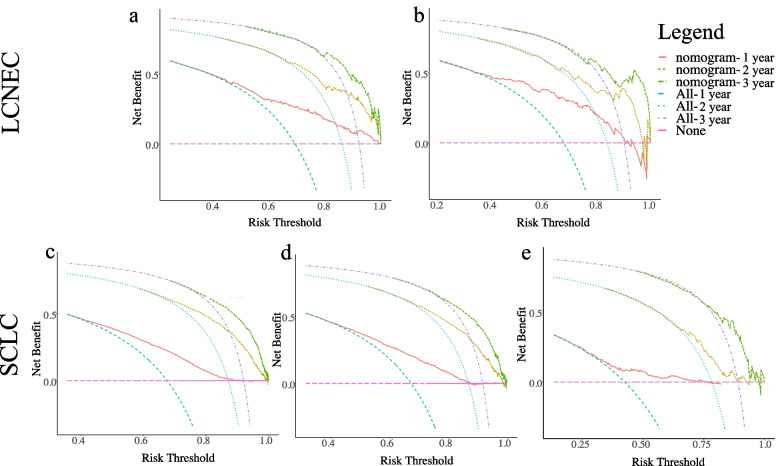
The DCA plots of the training cohort and validation cohort of LCNEC (**a**-**b**) and SCLC (**c**-**d**), and the external validation cohort of SCLC (**e**). Abbreviation: DCA, decision curve analysis; LCNEC, large cell neuroendocrine carcinoma; SCLC, small cell lung cancer. Note: “All” refers to intervention for all, and “None” refers to intervention for none. Intervention is considered to be any behavioral or external factor considered by high-risk patients when obtaining positive results from the model

To validate the accuracy of the two established nomograms for stage III-IV LCNEC and SCLC, we applied three methods including calibration plots, ROC curves, as well as DCA curves. Figure 6 and Additional file 3. Supplementary Fig. 1 presented the calibration plots of the nomograms of LCNEC and SCLC in the training and validation cohorts, all of which revealed the favorable survival predictive accuracy of the nomograms at three different time points. Figure 7 displayed the 1-, 2-, and 3-year ROC curves of stage III-IV LCNEC and SCLC. In external validation of the SCLC nomogram with a Chinese cohort, the AUCs of 1-year, 2-year and 3-year ROC were 0.652, 0.669, and 0.750, respectively. Furthermore, to compare the prognostic value of our nomograms to the TNM staging system, we displayed the 3-year variable-dependent ROC curves of stage III-IV LCNEC and SCLC (Fig. 8). Additional file 3. Supplementary Fig. 2-3 displayed the 1-year and 2-year variable-dependent ROC curves. All the results of 1-, 2-, 3- year variable-dependent ROC curves verified the superior prognostic value of our nomograms for LCNEC and SCLC over the traditional T/N/M staging system. Additionally, the DCA analyses also illustrated that the nomogram for 1-, 2-, and 3-year survival prediction of stage III-IV LCNEC and SCLC achieved robust positive net clinical benefits with a wide range of covered threshold probabilities (Fig. 9).

## Discussion

Tremendous progress has been made in the treatment of NSCLC, while limited progress has been approached in the field of clinical and molecular features as well as personalized therapy of lung high-grade neuroendocrine neoplasms, especially LCNEC. Neuroendocrine lung carcinomas represent around 20% of lung cancer and comprises SCLC (15%), large cell neuroendocrine carcinoma (LCNEC) (3%), typical carcinoids (TCs) (1.8%), and atypical carcinoids (ACs) (0.2%) [12–14]. Among them, LCNEC and SCLC belong to high-grade neuroendocrine lung carcinoma with poor prognosis. In our analysis, the incidence of LCNEC has increased over the last two decades, which might be partly because of the better recognition of this subtype entity [15]. As shown in Fig. 2, the incidence of LCNEC remains zero until 2000. It’s likely that cases of LCNEC might be underestimated and were not accurately identified because prior to 1999, the World Health Organization (WHO) classified LCNEC as a subtype of large cell carcinoma, while since 1999, WHO classification has been revised to officially recognize LCNEC as a separate entity from other types of lung cancer [10]. Conversely, the incidence of SCLC and other types of NSCLC were both decreased over years since 1990, which was consist with previous studies [16]. Reduced rate of cigarette smoking might partly account for the decreased rates of SCLC and SCC. Unlike NSCLC, the survival of LCNEC and SCLC failed to improved satisfyingly due to the limited advance in treatment in recent years [16].

The pathology histology diagnosis of LCNEC is a challenging mission even for pathologists when adopting the current WHO criteria for LCNEC diagnosis [17]. Two phase II studies showed that approximately 25% of recruited LCNEC patients turned out to be SCLC or NSCLC after central pathological review [18, 19]. The accuracy of LCNEC diagnosis in real-world might be even lower. Fortunately, further combining criteria regarding morphology, immunohistochemistry and molecular characteristics to refine the diagnosis of LCNEC is on the way [17].

The results of our analysis revealed that the survival outcome of stage III-IV LCNEC and SCLC represented similar survival outcome both in multivariate Cox analysis of the whole lung cancer patients and direct comparison after balancing several potential covariates including age, sex, race, bilaterality, TNM stage, organ metastases, and treatment strategies using PSM method. The findings are consistent with previous study results. Kinslow et al. proposed that stage I-III LCNEC behaved similarly to NSCLC, whereas stage IV LCNEC was more akin to SCLC [7]. Wang et al. proposed that better survival outcomes were observed in patients with LCNEC than SCLC in the subgroup of regional, distant, and surgery population, while in non-surgery subgroup, survival difference was not caught [20]. In other studies, the OS of patients with advanced LCNEC resembles that of SCLC [7, 21].

To date, the standard of care for LCNEC has not reached a consensus. For resectable LCNEC, surgical excision is recommended even though there was a high risk of recurrence after surgery alone. For those in locally advanced or metastatic stage, no standard treatment has been recognized [19]. Both the SCLC-type chemotherapy regimens and NSCLC-type agents were utilized in clinical practice based on the preference of clinicians, while the responses were unsatisfying. LCNEC was found to be more aggressive than other types of NSCLC and less responsive to SCLC-type chemotherapy regimens [22, 23]. Recently immune checkpoint inhibitors (ICIs) treatment showed promising antitumor activity against LCNEC [24]. In a recent meta-analysis, the pooled objective response rates (ORRs) of ICIs treatment for neuroendocrine neoplasms (NENs) from any sites, thoracic NENs, and pulmonary LCNEC were 15.5% (95% CI, 9.5–24.3%), 24.7% (95% CI, 16.1–36.1%), and 34.2% (95% CI, 20.3–51.6%), respectively [25]. For limited-stage SCLC, simultaneous radiation and chemotherapy would be the standard treatment strategy, whereas for extensive-stage SCLC, chemotherapy (etoposide plus platinum) combined with a programmed cell death-ligand 1 (PD-L1) antibody is recognized as the first-line therapy currently [26, 27]. Surgery is not recommended for patients with SCLC. Just like what we presented here, surgery was conducted in 13.4% and 1.3% of patients with stage III-IV LCNEC or SCLC, respectively.

In this study, apart from TNM stage, several other pretreatment factors like senior patients (over 70 years old) and distant organ metastasis were deemed as independent risk factors for both LCNEC and SCLC and were included in our final nomograms. The results were consistent with previous literatures [5, 7]. In terms of metastatic sites, bone, liver, and brain metastasis exerted independent adverse impacts on both of the two types of neuroendocrine lung carcinoma. We also found different metastatic patterns in patients with LCNEC and those with SCLC. LCNEC patients were observed to undertake a higher risk of brain metastasis, while SCLC were more frequently to suffer liver metastasis. Those finding were consistent with other studies [11, 20, 28]. Besides, bone was found to be the most common metastatic site of lung cancer among the four metastatic sites, and the incidence of bone metastasis in LCNEC, SCLC, and other NSCLC were all above 20% in this study. Of note, bone metastasis could produce different level of body pain, pathologic fracture, or even spinal cord compression and other morbidity, which could massively deteriorate patient quality of life [29]. The prognostic significance of bone metastasis in SCLC, especially bone multi-metastasis was also confirmed in our previous studies [5]. Since SCLC and LCNEC are prone to bone metastasis, physicians should be paying close attention to patient related symptoms and try local treatment such as stereotactic radiotherapy for oligometastasis when it’s still applicable [30]. Lung metastasis failed to show independent association with the OS of LCNEC in our data, but lung metastasis and bilaterality was associated with poor survival of patients of SCLC. In this study, two separate nomograms regarding patients with stage III-IV LCNEC or SCLC were established with favorable accuracy, the nomogram of SCLC was extra validated by an external Chinese cohort. By comparing the AUC of the ROC curves of our newly developed nomograms with T/N/M stage, our nomograms presented superior predictive accuracy than T/N/M stage. The superiority is especially shown in the Chinese SCLC cohort, which indicate the newly developed nomograms of SCLC may have even better application value in real world. We also respectively categorized patients with stage III-IV LCNEC or SCLC into two risk-subgroups based on the scores of nomograms, besides the online tools we designed can easily calculate the score and tell us the risk group of an individual patient, which might help clinicians better evaluate the severity of disease and apply nomograms into clinical practice.

In this study, we explored the incidence, survival comparison of stage III-IV LCNEC and SCLC, and created novel prognostic evaluation approaches for them. While several limitations existed in this study. Firstly, the pathology histologic diagnoses for SCLC and LCNECs were dependent on the ICD-O-3 record of SEER database, the accuracy of the pathological diagnoses cannot be double verified by other experts since the tissue slides were not accessible to us. Secondly, some other potential prognostic indexes such as molecular characteristic like key staining of neuroendocrine markers and standard driver oncogenes of LCNEC, as well as the detailed chemotherapeutic regimens, were not provided in SEER database, the value of those factors need to be investigated in further study with more detailed clinical and molecular data. Thirdly, to avoid the error caused by incongruous stage system, we only included patients involving data of stage based on 7th version of AJCC TNM stage [31]. In the current 8th version of TNM stage, stage T3 and T4 were defined differently from the 7th version, while N and M stage were consistent in the general direction [32]. Further studies might need to identify the prognostic significance of our prognostic models under the 8th version of AJCC TNM stage.

## Conclusions

In summary, our analysis revealed epidemiological trends and survival outcomes between locally advanced or metastatic LCNEC, SCLC, and other NSCLC based on large sample-based cohort. Furthermore, two novel prognostic evaluation approaches respectively for LCNEC and SCLC might present as practical tools for clinicians to predict the survival outcome of those patients and facilitate risk stratification.

## Supplementary Information

**Additional file 1.**

**Additional file 2.**

**Additional file 3.**

## Data Availability

Data source: https://seer.cancer.gov/. The data of external cohort of SCLC can be accessible upon reasonable request.

## Abbreviations

LCNEC: Large cell neuroendocrine carcinoma
SCLC: Small cell lung cancer
NSCLC: Non-small cell lung cancer
TC: Typical carcinoid
ADC: Adenocarcinoma
SCC: Squamous cell carcinoma
LCC: NOS: large cell carcinoma, not otherwise specified
AC: Atypical carcinoid
SEER: Surveillance, Epidemiology and End Results
PSM: Propensity score matching
WHO: World Health Organization
OS: Overall survival
AJCC: American Joint Committee on Cancer
APC: Annual percentage changes
DCA: Decision curve analysis
C-index: Concordance index
HR: Hazard ratio
CI: Confidence interval
SCC: Squamous cell carcinoma
ROC: Receiver operating characteristic
ICIs: Immune checkpoint inhibitors
ORR: Objective response rate
NENs: Neuroendocrine neoplasms
PD-L1: Programmed cell death-ligand 1
