# Supplementary material for: Incidence, survival comparison, and novel prognostic evaluation approaches for stage iii-iv pulmonary large cell neuroendocrine carcinoma and small cell lung cancer
Source: BMC Cancer. 2023 Apr 5;23:312. doi: 10.1186/s12885-023-10797-3 (PMC10077650; doi:10.1186/s12885-023-10797-3)
Supplement: Supplementary file 1 — Additional file 1. [file 12885_2023_10797_MOESM1_ESM.zip › Supplementary file 1/Study Instruction Manual.docx]

**Study Instruction Manual**

1. **Data acquisition**

**1.1 Software and account**

The SEER data was extracted using SEER Stat software (version 8.4.0, SEER ID: 12834-Nov2021).

**1.2 The cohort for analyzing the trend of long-term age-adjusted incidence of lung cancer.**

***Database Name***: Incidence - SEER Research Data, 8 Registries, Nov 2021 Sub (1975-2019)

***Statistics***: Trends (Age-Adjusted)

***Selection***:


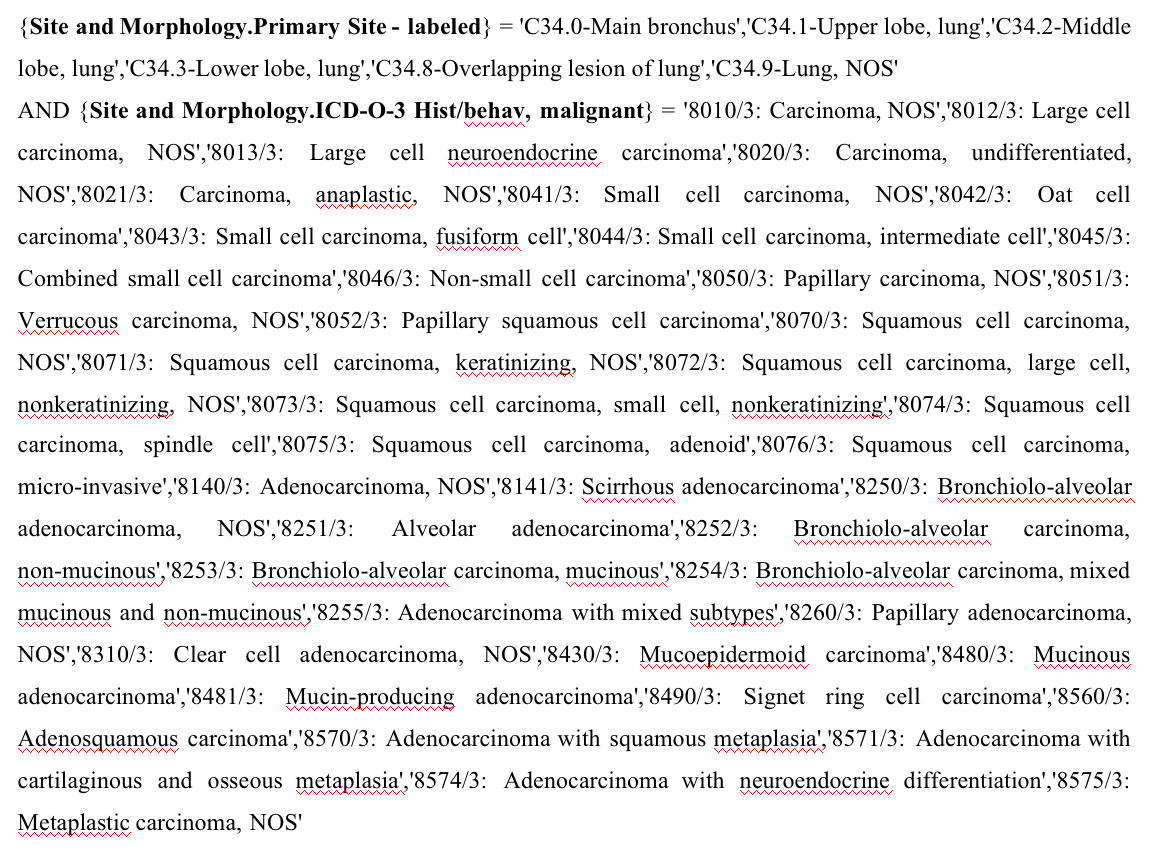


**1.3 The cohort for analyzing the prognosis and risk factors of LCNEC and SCLC.**

***Database Name***: Incidence - SEER Research Plus Data, 17 Registries, Nov 2021 Sub (2000-2019)

***Selection***: Use the same criteria of {Site and Morphology.Primary Site - labeled} and {Site and Morphology.ICD-O-3 Hist/behav, malignant} as mentioned in the selection part of 1.2, and add one another criterion as below:

{Year of diagnosis} = '2010','2011','2012','2013','2014','2015'

***Table*** *(columns that selected)*:


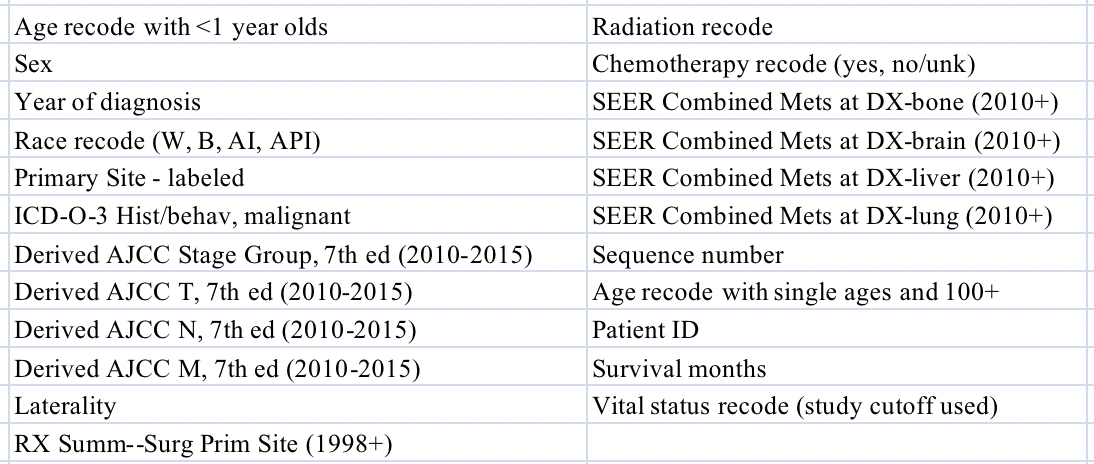


Note: 1) the column “RX Summ--Surg Prim Site (1998+)” is the surgery record: code 20-90 were set as “Yes”, others were set as “No/unknown”;

1. the column “Radiation recode” is the radiation recode: “Beam radiation/Combination of beam with implants or isotopes/Radiation, NOS method or source not specified/Radioactive implants (includes brachytherapy) (1988+)/Radioisotopes (1988+)” were set as “Yes”, others were set as “No/unknown”;
2. the column “Laterality” was used to create a column “Bilaterality”: “Bilateral, single primary” was set as “Yes”, others were set as “No”.

**2. Data filtration and study diagram**

**3. Data deposit.**

The SEER data analyzed in this study is be downloaded in Supplementary file 2.
