# Supplementary material for: Incidence, survival comparison, and novel prognostic evaluation approaches for stage iii-iv pulmonary large cell neuroendocrine carcinoma and small cell lung cancer
Source: BMC Cancer. 2023 Apr 5;23:312. doi: 10.1186/s12885-023-10797-3 (PMC10077650; doi:10.1186/s12885-023-10797-3)
Supplement: Supplementary file 3 — Additional file 3. [file 12885_2023_10797_MOESM3_ESM.docx]

**Figure S1.** The 1-year, 2-year, and 3-year calibration plots of internal validation cohort of LCNEC (a-c) and SCLC (d-f), and external validation cohort of SCLC (g-i).

Abbreviation: LCNEC, large cell neuroendocrine carcinoma; SCLC, small cell lung cancer.

**Figure S2.** The 1-year variable-dependent ROC curves of the training cohort and validation cohort of LCNEC (a-b) and SCLC (c-d), and the external validation cohort of SCLC (e).

**Figure S3.** The 2-year variable-dependent ROC curves of the training cohort and validation cohort of LCNEC (a-b) and SCLC (c-d), and the external validation cohort of SCLC (e).

**Supplementary Table 1.** Univariate and multivariate Cox analysis of all patients.

| **Variables** | **Univariate analysis** | | **Multivariate analysis** | |
| --- | --- | --- | --- | --- |
|  | **HR (95% CI)** | **P** | **HR (95% CI)** | **P** |
| **Age** |  |  |  |  |
| ≥70 vs ＜70 | 1.02(1.01-1.02) | **<0.001** | 1.02(1.02-1.02) | **<0.001** |
| **Sex** |  |  |  |  |
| Male vs Female | 1.19(1.17-1.2) | **<0.001** | 1.19(1.18-1.21) | **<0.001** |
| **Race** |  |  |  |  |
| Others vs Black | 0.79(0.77-0.82) | **<0.001** | 0.74(0.72-0.76) | **<0.001** |
| White vs Black | 1.01(0.99-1.03) | 0.428 | 0.97(0.95-0.99) | **0.001** |
| **Histology** |  |  |  |  |
| ADC vs LCNEC | 0.79(0.74-0.85) | **<0.001** | 0.74(0.69-0.8) | **<0.001** |
| SCC vs LCNEC | 0.9(0.84-0.97) | **0.005** | 0.91(0.84-0.98) | **0.008** |
| LCC,NOS vs LCNEC | 1.01(0.91-1.12) | 0.914 | 1(0.91-1.11) | 0.939 |
| SCLC vs LCNEC | 1(0.93-1.07) | 0.926 | 0.93(0.87-1) | 0.057 |
| NSCLC,NOS vs LCNEC | 1.08(1-1.16) | **0.045** | 1.01(0.94-1.09) | 0.812 |
| Others vs LCNEC | 1.17(1.08-1.26) | **<0.001** | 1.07(0.99-1.15) | 0.104 |
| **T stage** |  |  |  |  |
| T1 vs T0 | 0.93(0.86-1) | 0.051 | 1.03(0.96-1.12) | 0.37 |
| T2 vs T0 | 1.13(1.05-1.21) | **0.001** | 1.21(1.12-1.3) | **<0.001** |
| T3 vs T0 | 1.22(1.13-1.31) | **<0.001** | 1.29(1.2-1.39) | **<0.001** |
| T4 vs T0 | 1.25(1.16-1.34) | **<0.001** | 1.33(1.24-1.43) | **<0.001** |
| **N stage** |  |  |  |  |
| N1 vs N0 | 0.95(0.93-0.98) | **0.001** | 1.09(1.05-1.12) | **<0.001** |
| N2 vs N0 | 1.01(1-1.03) | 0.111 | 1.24(1.22-1.27) | **<0.001** |
| N3 vs N0 | 1.15(1.13-1.17) | **<0.001** | 1.3(1.27-1.33) | **<0.001** |
| **M stage** |  |  |  |  |
| M1 vs M0 | 1.9(1.87-1.93) | **<0.001** | 2.08(2.05-2.11) | **<0.001** |
| **Stage** |  |  |  |  |
| IV vs III | 1.9(1.87-1.93) | **<0.001** | - | - |
| **Bilaterality** |  |  |  |  |
| Yes vs No | 1.3(1.23-1.38) | **<0.001** | 1(0.94-1.06) | 0.957 |

Abbreviation: LCNEC, large cell neuroendocrine carcinoma; ADC, adenocarcinoma; SCC, squamous cell carcinoma; LCC,NOS, large cell carcinoma, not otherwise specified; SCLC, small cell lung cancer; NSCLC, non-small cell lung cancer; HR, hazard ratio; CI, confidence interval.

**Supplementary Table 2.** Comparison of LCNEC and SCLC before and after PSM analysis.

| **Variables** | **Before PSM** | | | **After PSM** | | |
| --- | --- | --- | --- | --- | --- | --- |
|  | **LCNEC (%)** | **SCLC (%)** | **P** | **LCNEC (%)** | **SCLC (%)** | **P** |
|  | **(n=785)** | **(n=15776)** |  | **(n=785)** | **(n=1570)** |  |
| **Age** |  |  | 0.013 |  |  | 0.815 |
| <70 | 535 (68.2%) | 10051 (63.7%) |  | 535 (68.2) | 1061 (67.6) |  |
| ≥70 | 250 (31.8%) | 5725 (36.3%) |  | 250 (31.8) | 509 (32.4) |  |
| **Sex** |  |  | <0.001 |  |  | 1 |
| Female | 331 (42.2%) | 7859 (49.8%) |  | 331 (42.2) | 662 (42.2) |  |
| Male | 454 (57.8%) | 7917 (50.2%) |  | 454 (57.8) | 908 (57.8) |  |
| **Race** |  |  | 0.002 |  |  | 0.859 |
| Black | 102 (13.0%) | 1451 (9.2%) |  | 102 (13.0) | 203 (12.9) |  |
| Others | 31 (3.9%) | 719 (4.6%) |  | 31 (3.9) | 55 (3.5) |  |
| White | 652 (83.1%) | 13606 (86.2%) |  | 652 (83.1) | 1312 (83.6) |  |
| **Stage** |  |  | 0.567 |  |  | 0.962 |
| III | 228 (29.0%) | 4744 (30.1%) |  | 228 (29.0) | 459 (29.2) |  |
| IV | 557 (71.0%) | 11032 (69.9%) |  | 557 (71.0) | 1111 (70.8) |  |
| **T stage** |  |  | <0.001 |  |  | 0.972 |
| T0 | 11 (1.4%) | 207 (1.3%) |  | 11 (1.4) | 17 (1.1) |  |
| T1 | 113 (14.4%) | 1667 (10.6%) |  | 113 (14.4) | 227 (14.5) |  |
| T2 | 220 (28.0%) | 3895 (24.7%) |  | 220 (28.0) | 439 (28.0) |  |
| T3 | 185 (23.6%) | 3523 (22.3%) |  | 185 (23.6) | 366 (23.3) |  |
| T4 | 256 (32.6%) | 6484 (41.1%) |  | 256 (32.6) | 521 (33.2) |  |
| **N stage** |  |  | <0.001 |  |  | 0.962 |
| N0 | 155 (19.7%) | 1572 (10.0%) |  | 155 (19.7) | 322 (20.5) |  |
| N1 | 69 (8.8%) | 949 (6.0%) |  | 69 (8.8) | 131 (8.3) |  |
| N2 | 395 (50.3%) | 9431 (59.8%) |  | 395 (50.3) | 789 (50.3) |  |
| N3 | 166 (21.1%) | 3824 (24.2%) |  | 166 (21.1) | 328 (20.9) |  |
| **M stage** |  |  | 0.567 |  |  | 0.962 |
| M0 | 228 (29.0%) | 4744 (30.1%) |  | 228 (29.0) | 459 (29.2) |  |
| M1 | 557 (71.0%) | 11032 (69.9%) |  | 557 (71.0) | 1111 (70.8) |  |
| **Bilaterality** |  |  | 0.873 |  |  | 0.201 |
| No | 778 (99.1%) | 15654 (99.2%) |  | 778 (99.1) | 1564 (99.6) |  |
| Yes | 7 (0.9%) | 122 (0.8%) |  | 7 (0.9) | 6 (0.4) |  |
| **Bone metastasis** |  |  | 0.175 |  |  | 0.875 |
| No | 606 (77.2%) | 11830 (75.0%) |  | 606 (77.2) | 1218 (77.6) |  |
| Yes | 179 (22.8%) | 3946 (25.0%) |  | 179 (22.8) | 352 (22.4) |  |
| **Liver metastasis** |  |  | <0.001 |  |  | 0.844 |
| No | 617 (78.6%) | 11231 (71.2%) |  | 617 (78.6) | 1241 (79.0) |  |
| Yes | 168 (21.4%) | 4545 (28.8%) |  | 168 (21.4) | 329 (21.0) |  |
| **Lung metastasis** |  |  | 0.974 |  |  | 0.46 |
| No | 670 (85.4) | 13482 (85.5) |  | 670 (85.4) | 1359 (86.6) |  |
| Yes | 115 (14.6) | 2294 (14.5) |  | 115 (14.6) | 211 (13.4) |  |
| **Brain metastasis** |  |  | <0.001 |  |  | 0.987 |
| No | 564 (71.8%) | 12843 (81.4%) |  | 564 (71.8) | 1130 (72.0) |  |
| Yes | 221 (28.2%) | 2933 (18.6%) |  | 221 (28.2) | 440 (28.0) |  |
| **Surgery (%)** |  |  |  |  |  | 0.555 |
| No/Unknown | 680 (86.6) | 15575 (98.7) | <0.001 | 680 (86.6) | 1375 (87.6) |  |
| Yes | 105 (13.4) | 201 (1.3) |  | 105 (13.4) | 195 (12.4) |  |
| **Chemotherapy (%)** |  |  |  |  |  | 0.321 |
| No/Unknown | 228 (29.0) | 2914 (18.5) | <0.001 | 228 (29.0) | 424 (27.0) |  |
| Yes | 557 (71.0) | 12862 (81.5) |  | 557 (71.0) | 1146 (73.0) |  |
| **Radiation (%)** |  |  |  |  |  | 0.965 |
| No/Unknown | 333 (42.4) | 7143 (45.3) | 0.125 | 333 (42.4) | 669 (42.6) |  |
| Yes | 452 (57.6) | 8633 (54.7) |  | 452 (57.6) | 901 (57.4) |  |

Abbreviation: LCNEC, large cell neuroendocrine carcinoma; SCLC, small cell lung cancer; PSM, propensity score matching.

**Supplementary Table 3.** Comparison of LCNEC and other NSCLC before and after PSM analysis.

| **Variables** | **Before PSM** | | | **After PSM** | | |
| --- | --- | --- | --- | --- | --- | --- |
|  | **LCNEC (%)** | **NSCLC (%)** | **P** | **LCNEC (%)** | **NSCLC (%)** | **P** |
|  | **(n=785)** | **(n=75074)** |  | **(n=785)** | **(n=1570)** |  |
| **Age** |  |  | <0.001 |  |  | 0.913 |
| <70 | 535 (68.2%) | 42718 (56.9%) |  | 535 (68.2) | 1065 (67.8) |  |
| ≥70 | 250 (31.8%) | 32356 (43.1%) |  | 250 (31.8) | 505 (32.2) |  |
| **Sex** |  |  | 0.147 |  |  | 0.988 |
| Female | 331 (42.2%) | 33645 (44.8%) |  | 331 (42.2) | 664 (42.3) |  |
| Male | 454 (57.8%) | 41429 (55.2%) |  | 454 (57.8) | 906 (57.7) |  |
| **Race** |  |  | <0.001 |  |  | 0.994 |
| Black | 102 (13.0%) | 9651 (12.9%) |  | 102 (13.0) | 202 (12.9) |  |
| Others | 31 (3.9%) | 6945 (9.3%) |  | 31 (3.9) | 63 (4.0) |  |
| White | 652 (83.1%) | 58478 (77.9%) |  | 652 (83.1) | 1305 (83.1) |  |
| **Stage** |  |  | 0.051 |  |  | 1 |
| III | 228 (29.0%) | 24316 (32.4%) |  | 228 (29.0) | 456 (29.0) |  |
| IV | 557 (71.0%) | 50758 (67.6%) |  | 557 (71.0) | 1114 (71.0) |  |
| **T stage** |  |  | 0.029 |  |  | 0.999 |
| T0 | 11 (1.4%) | 624 (0.8%) |  | 11 (1.4) | 20 (1.3) |  |
| T1 | 113 (14.4%) | 8685 (11.6%) |  | 113 (14.4) | 229 (14.6) |  |
| T2 | 220 (28.0%) | 20667 (27.5%) |  | 220 (28.0) | 437 (27.8) |  |
| T3 | 185 (23.6%) | 19624 (26.1%) |  | 185 (23.6) | 367 (23.4) |  |
| T4 | 256 (32.6%) | 25474 (33.9%) |  | 256 (32.6) | 517 (32.9) |  |
| **N stage** |  |  | 0.826 |  |  | 1 |
| N0 | 155 (19.7%) | 14948 (19.9%) |  | 155 (19.7) | 311 (19.8) |  |
| N1 | 69 (8.8%) | 6363 (8.5%) |  | 69 (8.8) | 139 (8.9) |  |
| N2 | 395 (50.3%) | 38767 (51.6%) |  | 395 (50.3) | 788 (50.2) |  |
| N3 | 166 (21.1%) | 14996 (20.0%) |  | 166 (21.1) | 332 (21.1) |  |
| **M stage** |  |  | 0.051 |  |  | 1 |
| M0 | 228 (29.0%) | 24316 (32.4%) |  | 228 (29.0) | 456 (29.0) |  |
| M1 | 557 (71.0%) | 50758 (67.6%) |  | 557 (71.0) | 1114 (71.0) |  |
| **Bilaterality** |  |  | 0.303 |  |  | 0.297 |
| No | 778 (99.1%) | 74032 (98.6%) |  | 778 (99.1) | 1563 (99.6) |  |
| Yes | 7 (0.9%) | 1042 (1.4%) |  | 7 (0.9) | 7 (0.4) |  |
| **Bone metastasis** |  |  | 0.057 |  |  | 0.986 |
| No | 606 (77.2%) | 55663 (74.1%) |  | 606 (77.2) | 1214 (77.3) |  |
| Yes | 179 (22.8%) | 19411 (25.9%) |  | 179 (22.8) | 356 (22.7) |  |
| **Liver metastasis** |  |  | <0.001 |  |  | 0.986 |
| No | 617 (78.6%) | 66924 (89.1%) |  | 617 (78.6) | 1232 (78.5) |  |
| Yes | 168 (21.4%) | 8150 (10.9%) |  | 168 (21.4) | 338 (21.5) |  |
| **Lung metastasis** |  |  | <0.001 |  |  | 0.984 |
| No | 670 (85.4) | 58797 (78.3) |  | 670 (85.4) | 1342 (85.5) |  |
| Yes | 115 (14.6) | 16277 (21.7) |  | 115 (14.6) | 228 (14.5) |  |
| **Brain metastasis** |  |  | <0.001 |  |  | 0.91 |
| No | 564 (71.8%) | 61145 (81.4%) |  | 564 (71.8) | 1133 (72.2) |  |
| Yes | 221 (28.2%) | 13929 (18.6%) |  | 221 (28.2) | 437 (27.8) |  |
| **Surgery (%)** |  |  |  |  |  | 0.469 |
| No/Unknown | 680 (86.6) | 68677 (91.5) | <0.001 | 680 (86.6) | 1378 (87.8) |  |
| Yes | 105 (13.4) | 6397 (8.5) |  | 105 (13.4) | 192 (12.2) |  |
| **Chemotherapy (%)** |  |  |  |  |  | 0.987 |
| No/Unknown | 228 (29.0) | 27863 (37.1) | <0.001 | 228 (29.0) | 458 (29.2) |  |
| Yes | 557 (71.0) | 47211 (62.9) |  | 557 (71.0) | 1112 (70.8) |  |
| **Radiation (%)** |  |  |  |  |  | 0.988 |
| No/Unknown | 333 (42.4) | 35311 (47.0) | 0.011 | 333 (42.4) | 668 (42.5) |  |
| Yes | 452 (57.6) | 39763 (53.0) |  | 452 (57.6) | 902 (57.5) |  |

Abbreviation: LCNEC, large cell neuroendocrine carcinoma; NSCLC, non-small cell lung cancer; PSM, propensity score matching.

**Supplementary Table 4.** Training and validation cohort of stage III-IV LCNEC patients.

| **Variables** | **Training Cohort** | **Validation Cohort** | **Overall** |
| --- | --- | --- | --- |
|  | **(N=552)** | **(N=233)** | **(N=785)** |
| **Age** |  |  |  |
| <70 | 374 (67.8%) | 161 (69.1%) | 535 (68.2%) |
| ≥70 | 178 (32.2%) | 72 (30.9%) | 250 (31.8%) |
| **Sex** |  |  |  |
| Female | 240 (43.5%) | 91 (39.1%) | 331 (42.2%) |
| Male | 312 (56.5%) | 142 (60.9%) | 454 (57.8%) |
| **Race** |  |  |  |
| Black | 74 (13.4%) | 28 (12.0%) | 102 (13.0%) |
| Others | 18 (3.3%) | 13 (5.6%) | 31 (3.9%) |
| White | 460 (83.3%) | 192 (82.4%) | 652 (83.1%) |
| **Stage** |  |  |  |
| III | 161 (29.2%) | 67 (28.8%) | 228 (29.0%) |
| IV | 391 (70.8%) | 166 (71.2%) | 557 (71.0%) |
| **T stage** |  |  |  |
| T0 | 9 (1.6%) | 2 (0.9%) | 11 (1.4%) |
| T1 | 71 (12.9%) | 42 (18.0%) | 113 (14.4%) |
| T2 | 163 (29.5%) | 57 (24.5%) | 220 (28.0%) |
| T3 | 134 (24.3%) | 51 (21.9%) | 185 (23.6%) |
| T4 | 175 (31.7%) | 81 (34.8%) | 256 (32.6%) |
| **N stage** |  |  |  |
| N0 | 117 (21.2%) | 38 (16.3%) | 155 (19.7%) |
| N1 | 47 (8.5%) | 22 (9.4%) | 69 (8.8%) |
| N2 | 278 (50.4%) | 117 (50.2%) | 395 (50.3%) |
| N3 | 110 (19.9%) | 56 (24.0%) | 166 (21.1%) |
| **M stage** |  |  |  |
| M0 | 161 (29.2%) | 67 (28.8%) | 228 (29.0%) |
| M1 | 391 (70.8%) | 166 (71.2%) | 557 (71.0%) |
| **Bilaterality** |  |  |  |
| No | 547 (99.1%) | 231 (99.1%) | 778 (99.1%) |
| Yes | 5 (0.9%) | 2 (0.9%) | 7 (0.9%) |
| **Bone metastasis** |  |  |  |
| No | 439 (79.5%) | 167 (71.7%) | 606 (77.2%) |
| Yes | 113 (20.5%) | 66 (28.3%) | 179 (22.8%) |
| **Liver metastasis** |  |  |  |
| No | 439 (79.5%) | 178 (76.4%) | 617 (78.6%) |
| Yes | 113 (20.5%) | 55 (23.6%) | 168 (21.4%) |
| **Lung metastasis** |  |  |  |
| No | 478 (86.6%) | 192 (82.4%) | 670 (85.4%) |
| Yes | 74 (13.4%) | 41 (17.6%) | 115 (14.6%) |
| **Brain metastasis** |  |  |  |
| No | 391 (70.8%) | 173 (74.2%) | 564 (71.8%) |
| Yes | 161 (29.2%) | 60 (25.8%) | 221 (28.2%) |
| **Surgery** |  |  |  |
| No/Unknown | 485 (87.9%) | 195 (83.7%) | 680 (86.6%) |
| Yes | 67 (12.1%) | 38 (16.3%) | 105 (13.4%) |
| **Chemotherapy** |  |  |  |
| No/Unknown | 166 (30.1%) | 62 (26.6%) | 228 (29.0%) |
| Yes | 386 (69.9%) | 171 (73.4%) | 557 (71.0%) |
| **Radiation** |  |  |  |
| No/Unknown | 231 (41.8%) | 102 (43.8%) | 333 (42.4%) |
| Yes | 321 (58.2%) | 131 (56.2%) | 452 (57.6%) |

Abbreviation: LCNEC, large cell neuroendocrine carcinoma.

**Supplementary Table 5**. Training, validation and extra validation cohort of stage III-IV SCLC patients.

| **Variables** | **Training Cohort** | **Validation Cohort** | **Overall** | **Extra Validation Cohort** |
| --- | --- | --- | --- | --- |
|  | (N=11044) | (N=4732) | (N=15776) | (N=349) |
| **Age** |  |  |  |  |
| <70 | 7052 (63.9%) | 2999 (63.4%) | 10051 (63.7%) | 303 (86.8%) |
| ≥70 | 3992 (36.1%) | 1733 (36.6%) | 5725 (36.3%) | 46 (13.2%) |
| **Sex** |  |  |  |  |
| Female | 5434 (49.2%) | 2425 (51.2%) | 7859 (49.8%) | 68 (19.5%) |
| Male | 5610 (50.8%) | 2307 (48.8%) | 7917 (50.2%) | 281 (80.5%) |
| **Race** |  |  |  |  |
| Black | 1014 (9.2%) | 437 (9.2%) | 1451 (9.2%) | 0 (0%) |
| Others | 497 (4.5%) | 222 (4.7%) | 719 (4.6%) | 349 (100%) |
| White | 9533 (86.3%) | 4073 (86.1%) | 13606 (86.2%) |  |
| **Stage** |  |  |  |  |
| III | 3303 (29.9%) | 1441 (30.5%) | 4744 (30.1%) | 29 (8.3%) |
| IV | 7741 (70.1%) | 3291 (69.5%) | 11032 (69.9%) | 320 (91.7%) |
| **T stage** |  |  |  |  |
| T0 | 143 (1.3%) | 64 (1.4%) | 207 (1.3%) | 0 (0%) |
| T1 | 1161 (10.5%) | 506 (10.7%) | 1667 (10.6%) | 47 (13.5%) |
| T2 | 2740 (24.8%) | 1155 (24.4%) | 3895 (24.7%) | 100 (28.7%) |
| T3 | 2468 (22.3%) | 1055 (22.3%) | 3523 (22.3%) | 93 (26.6%) |
| T4 | 4532 (41.0%) | 1952 (41.3%) | 6484 (41.1%) | 109 (31.2%) |
| **N stage** |  |  |  |  |
| N0 | 1089 (9.9%) | 483 (10.2%) | 1572 (10.0%) | 8 (2.3%) |
| N1 | 665 (6.0%) | 284 (6.0%) | 949 (6.0%) | 122 (35.0%) |
| N2 | 6607 (59.8%) | 2824 (59.7%) | 9431 (59.8%) | 218 (62.5%) |
| N3 | 2683 (24.3%) | 1141 (24.1%) | 3824 (24.2%) | 1 (0.3%) |
| **M stage** |  |  |  |  |
| M0 | 3303 (29.9%) | 1441 (30.5%) | 4744 (30.1%) | 29 (8.3%) |
| M1 | 7741 (70.1%) | 3291 (69.5%) | 11032 (69.9%) | 320 (91.7%) |
| **Bilaterality** |  |  |  |  |
| No | 10963 (99.3%) | 4691 (99.1%) | 15654 (99.2%) | 318 (91.1%) |
| Yes | 81 (0.7%) | 41 (0.9%) | 122 (0.8%) | 31 (8.9%) |
| **Bone metastasis** |  |  |  |  |
| No | 8249 (74.7%) | 3581 (75.7%) | 11830 (75.0%) | 250 (71.6%) |
| Yes | 2795 (25.3%) | 1151 (24.3%) | 3946 (25.0%) | 99 (28.4%) |
| **Liver metastasis** |  |  |  |  |
| No | 7853 (71.1%) | 3378 (71.4%) | 11231 (71.2%) | 257 (73.6%) |
| Yes | 3191 (28.9%) | 1354 (28.6%) | 4545 (28.8%) | 92 (26.4%) |
| **Lung metastasis** |  |  |  |  |
| No | 9421 (85.3%) | 4061 (85.8%) | 13482 (85.5%) | 313 (89.7%) |
| Yes | 1623 (14.7%) | 671 (14.2%) | 2294 (14.5%) | 36 (10.3%) |
| **Brain metastasis** |  |  |  |  |
| No | 9002 (81.5%) | 3841 (81.2%) | 12843 (81.4%) | 289 (82.8%) |
| Yes | 2042 (18.5%) | 891 (18.8%) | 2933 (18.6%) | 60 (17.2%) |
| **Surgery** |  |  |  |  |
| No/Unknown | 10907 (98.8%) | 4668 (98.6%) | 15575 (98.7%) | 344 (98.6%) |
| Yes | 137 (1.2%) | 64 (1.4%) | 201 (1.3%) | 5 (1.4%) |
| **Chemotherapy** |  |  |  |  |
| No/Unknown | 2023 (18.3%) | 891 (18.8%) | 2914 (18.5%) | 57 (16.3%) |
| Yes | 9021 (81.7%) | 3841 (81.2%) | 12862 (81.5%) | 292 (83.7%) |
| **Radiation** |  |  |  |  |
| No/Unknown | 4988 (45.2%) | 2155 (45.5%) | 7143 (45.3%) | 189 (54.2%) |
| Yes | 6056 (54.8%) | 2577 (54.5%) | 8633 (54.7%) | 160 (45.8%) |

Abbreviation: SCLC, small cell lung cancer.
